# Role of nutrition in patients with coexisting chronic obstructive pulmonary disease and sarcopenia

**DOI:** 10.3389/fnut.2023.1214684

**Published:** 2023-08-08

**Authors:** Yayun Nan, Yuting Zhou, Ziyu Dai, Ting Yan, Pingping Zhong, Fufeng Zhang, Qiong Chen, Linlin Peng

**Affiliations:** ^1^Department of Ningxia Geriatrics Medical Center, Ningxia People’s Hospital, Yinchuan, China; ^2^Department of Geriatrics, Xiangya Hospital, Central South University, Changsha, China; ^3^National Clinical Research Center for Geriatric Disorders, Xiangya Hospital, Central South University, Changsha, China

**Keywords:** nutrition, chronic obstructive pulmonary disease, sarcopenia, skeletal muscle, dietary supplements, therapy

## Abstract

Chronic obstructive pulmonary disease (COPD) is one of the most common chronic diseases in the elderly population and is characterized by persistent respiratory symptoms and airflow obstruction. During COPD progression, a variety of pulmonary and extrapulmonary complications develop, with sarcopenia being one of the most common extrapulmonary complications. Factors that contribute to the pathogenesis of coexisting COPD and sarcopenia include systemic inflammation, hypoxia, hypercapnia, oxidative stress, protein metabolic imbalance, and myocyte mitochondrial dysfunction. These factors, individually or in concert, affect muscle function, resulting in decreased muscle mass and strength. The occurrence of sarcopenia severely affects the quality of life of patients with COPD, resulting in increased readmission rates, longer hospital admission, and higher mortality. In recent years, studies have found that oral supplementation with protein, micronutrients, fat, or a combination of nutritional supplements can improve the muscle strength and physical performance of these patients; some studies have also elucidated the possible underlying mechanisms. This review aimed to elucidate the role of nutrition among patients with coexisting COPD and sarcopenia.

## 1. Introduction

Chronic obstructive pulmonary disease (COPD) causes severe global burden of disease and socioeconomic burden and is a major cause of mortality worldwide. According to the Lancet 2019 Global Burden of Disease, COPD induced 74.4 million global Disability-Adjusted Life Years (DALYs) (for comparison: ischemic heart disease–182 million; stroke–143 million; tracheal, bronchus, and lung cancer–45.9 million; stomach cancer–22.2 million; liver cancer–12.5 million), and 71.9% of the DALYs are related to total chronic respiratory disease (1). COPD is often associated with various extrapulmonary pathologies; however, in the elderly, COPD often coexists with geriatric syndromes (GS), including sarcopenia, frailty, and malnutrition. Sarcopenia is a common GS characterized by progressive muscle mass loss associated with increased age and decreased muscle strength, with or without low physical performance. The clinical manifestations of sarcopenia include decreased muscle strength, mobility, and decreased gait speed. The prevalence of coexisting COPD and sarcopenia is 4.4–68% (Table 1) in different countries and regions.

**TABLE 1:** Characteristics of the included studies regarding the prevalence of coexisting chronic obstructive pulmonary disease and sarcopenia.

References	Country	Sample size *n*	Male *n* (%)	Age mean ± SD	Study design	Diagnosis of sarcopenia			Prevalence *n* (%)
						Muscle mass assessment	Muscle strength assessment	Physical performance assessment	
Tsekoura et al. (2)	Greece	69	10 (15%)	71.33 ± 7.48	cross-sectional study	BIA	HGS	4MGS	17 (24.6%)
Jones et al. (3)	UK	622	354 (57%)	NA	cross-sectional study	BIA	HGS	4MGS	90 (14.5%)
Kaluźniak-Szymanowska et al. (4)	Poland	124	74 (60%)	69.4 ± 6.1	cross-sectional study	BIA	The upper limb muscle strength:HGS The lower limb muscle strength: CST	6MWT	16 (12.9%)
Perrot et al. (5)	France	54	37 (69%)	68 ± 9	cross-sectional study	BIA	HGS	–	26 (48%)
Chung et al. (6)	Korea	1,039	760 (73%)	64.5 ± 9.4 (M) 64.5 ± 10.2 (F)	retrospective study	DXA	–	–	682 (34%)
Gologanu et al. (7)	Romania	36	12 (33%)	65.6 ± 7.5	cross-sectional study	BIA	–	6MWT	3 (8%)
Trajanoska et al. (8)	Netherlands	COPD: 882 All population: 5,911		69.2 ± 9.1	prospective study	DXA	HGS	GAITRite	COPD: 70 (7.9%) All population: 260 (4.4%)
Cebron Lipovec MPharm et al. (9)	Slovenia	112	74 (66%)	66 ± 8	prospective study	DXA	–	–	61 (54%)
Limpawattana et al. (10)	Thailand	121	112 (92.6%)	NA	cross-sectional study	DXA	–	6MWT	29 (24%)
Demircioğlu et al. (11)	Turkey	219	196 (89.5%)	66.9 ± 10.1	cross-sectional study	BIA	HGS	6MWT	97 (44.3%)
Persson et al. (12)	Sweden	32	13 (41%)	68 ± 6	cross-sectional study	BIA	–	–	21 (68%)

BIA, bioelectrical impedance analysis; HGS, handgrip strength; 4MGS, 4-metre gait speed; CST, chair stand test; 6MWT, the Six-Minute Walk Test; M, male; F, female; DXA, dual-energy X-ray absorptiometry; GAITRite, 5.79-m-long electronic walkway.

Patients with coexisting COPD and sarcopenia commonly have a markedly reduced fat-free mass index (FFMI) and typically present with reduced muscle size, muscle atrophy, and muscle fiber shift. Multiple multicenter studies with a large sample size have shown that, regardless of other factors, a low FFMI is a significant predictor of mortality in patients with moderate-to-severe COPD (13–16). In the elderly population, lower hand grip strength is significantly associated with moderate-to-very severe airflow limitation (Forced Expiratory Volume in the first second <80% of predicted value) (17). Therefore, muscle mass growth aimed at increasing FFMI is important for maintaining the quality of life and improving prognosis in patients with coexisting COPD and sarcopenia. Studies have shown that sarcopenia correlates with health status, quality of life, and survival, whereas low muscle strength is associated with an increased risk of death from cardiovascular disease, respiratory disease, and cancer (18–21). In recent years, studies have explored the decline of physical capacity in patients with coexisting COPD and sarcopenia, and have attempted to identify specific biomarkers. Additionally, other studies have sought to understand the pathogenesis of COPD and sarcopenia, including the interaction between COPD and sarcopenia, and the potential role of low nutritional intake and high energy consumption in patients with COPD and sarcopenia (22). Furthermore, some animal studies have attempted to explore the intrinsic mechanisms of sarcopenia and COPD comprehensively. Several studies have focused on providing nutritional support to patients with COPD and enrolling them in pulmonary rehabilitation programs to improve sarcopenia. These approaches have the potential to positively impact intrinsic capacity (23) and increase life expectancy in patients with coexisting COPD and sarcopenia. An appropriate nutritional support formulation, either single or compounded, can increase FFMI and improve muscle function and exercise performance in patients with COPD. A considerable amount of research has been conducted on the components of nutritional support with the aim to identify formulas that are beneficial to patients with coexisting COPD and sarcopenia and clarify the underlying mechanisms of muscle growth and function improvement. This review provides an overview of the role of nutrition among patients with coexisting COPD and sarcopenia.

## 2. Pathogenesis

### 2.1. Systemic inflammation

The elevated expression of multiple pro-inflammatory factors may affect the loss of skeletal muscle mass and function during COPD pathogenesis (24). When muscle wasting occurs, the expression levels of pro-inflammatory cytokines and their receptors are upregulated, leading to metabolic disturbances in muscle tissue (25), ultimately resulting in sarcopenia (26). Besides muscle wasting, patients with more severe COPD often have deposits of ectopic fat, including visceral adipose tissue, and muscle fat infiltration; both of which are associated with elevated markers of systemic inflammation (12). Clinical studies have found increased levels of interleukin (IL)-6, IL-10, hypersensitive C-reactive protein (CRP), and tumor necrosis factor (TNF)-α in patients with coexisting COPD and sarcopenia (27, 28). In animal study, it was observed that a decrease in muscle protein coincided with a sharp increase in pro-inflammatory cytokines (TNF-α, IL-6, and interferon-γ) (29). Further experiments showed that IL-6 can activate the Janus kinase/signal transducer and activator of the transcription (STAT) signaling pathway, with STAT transcriptional activation stimulating the expression and activity of CCAAT-enhancer-binding proteins (C/EBPδ). This in turn increases the expression and activity of myostatin (MSTN), muscle atrophy F-box gene (MAFbx/Atrogin-1), muscle ring finger 1 (MuRF1), and cysteine-aspartic proteases-3 (caspase-3) in muscle fibers to enhance muscle protein hydrolysis (30–32). TNF-α may inhibit the expression of selective MAFbx genic genes in late myogenesis by activating the mitogen-activated protein kinase (MAPK) pathway (33). Studies from different countries and regions have concluded that there is a positive association between a pro-inflammatory diet and sarcopenia (34–36). Moreover, they suggested that an anti-inflammatory diet can reduce the systemic inflammatory response and improve sarcopenia (37, 38). However, the mechanism by which the anti-inflammatory diet works needs to be further clarified. Further controlled clinical trials to determine the predictive or diagnostic role of pro-inflammatory cytokines in patients with coexisting COPD and sarcopenia are warranted.

### 2.2. Hypoxia and oxidative stress

The expression of sarcoplasmic reticulum (SR) stress markers, downstream apoptotic factors, and inflammatory factors is increased in patients with COPD (39). Compared with healthy controls, patients with advanced COPD have significantly lower SR Ca^2+^ ATPase activity (39, 40), smaller single muscle fiber diameters, and fewer cytoplasmic structural domains per myonucleus (39). These changes in SR dysfunction are accompanied by the generation of lipid peroxidation and mitochondrial reactive oxygen species (ROS), which indicate elevated levels of oxidative stress (41). Associated mechanisms may include elevated neural precursor cell expressed developmentally down-regulated protein (NEDD)4 expression in muscle (42) and transforming growth factor (TGF)-mediated MSTN (43). Nevertheless, there are contrasting hypotheses that a high CO_2_ environment can promote MSTN degradation by activating the AMPKα2-forkhead box O-like (FoxO) 3a-MuRF1 pathway (44, 45). Studies on the mechanisms of COPD combined with sarcopenia have shown high heterogeneity in the fields of hypoxia and oxidative stress; however, the sample sizes of the relevant clinical studies are relatively small. Notably, one study confirmed that oral supplementation of L-carnitine alleviates lipid peroxidation and biomarkers of muscle damage (46); however, this study was performed in healthy men, and it did not confirm whether the same effect was also present in patients with coexisting COPD and sarcopenia.

### 2.3. Mitochondrial dysfunction

In patients with COPD, skeletal muscle cells show reduced mitochondrial density and biosynthesis, reduced coupling of mitochondrial respiratory chain complexes, and increased mitochondrial ROS levels. Studies have found that patients with severe COPD (Global initiative for chronic obstructive lung disease 3/4) have significantly increased total ROS and mitochondrial ROS in quadriceps cells, as well as decreased mitochondrial membrane potential, superoxide dismutase 2, and electron transport chain (ETC) complex protein expression (47). Moreover, some studies have found an elevated expression of BCL2/adenovirus E1B 19 kDa protein-interacting protein 3–like, FUN14 domain containing 1 (FUNDC1), and parkin in the skeletal muscle of patients with COPD. These molecules promote mitochondrial catabolism in skeletal muscles (48). In rats with COPD, researchers observed decreased expression of PGC-1a and SIRT3, which is thought to be associated with skeletal muscle mitochondrial damage (49). Oral supplementation with branched-chain amino acids (50) and 12 weeks of progressive resistance exercise training (51) can improve physical performance in older adults by improving mitochondrial function. Most studies were observational and had certain limitations, including a small sample size. However, the specific molecular mechanisms underlying myocyte mitochondrial dysfunction in COPD with sarcopenia require further investigation.

### 2.4. Disorders of muscle catabolism and anabolism

During the development of COPD, signaling pathways that promote skeletal muscle growth are often inhibited, whereas those that promote skeletal muscle cell catabolism and anabolism are activated, resulting in abnormal muscle metabolism and negative changes in muscle strength and mass. MSTN, a member of the TGF-β family, is secreted by myoblasts and plays a negative role in muscle growth regulation. MSTN is significantly elevated in the serum of patients with COPD, suggesting that MSTN may be associated with the development of COPD and sarcopenia (41). Muscle growth can be induced by insulin-like growth factor (IGF), and mothers against decapentaplegic homolog 3 (MSTN-Smad3) plays a negative regulatory role in muscle growth. Some studies indicate that the molecular expression of the IGF-signaling pathway is enhanced after pulmonary rehabilitation training (52), whilst the molecular expression of the MSTN-Smad3 signaling pathway is attenuated (53). The IGF-1/protein kinase B (Akt)/FoxO signaling pathway affects muscle atrophy during aging. In various models of muscle atrophy, reduced Akt activity and phosphorylation levels of FoxO in the cytoplasm were observed, and the phosphorylation level of FoxO in the nucleus was significantly increased (54, 55). The inhibition of FoxO transcriptional activity suppresses MAFbx and MuRF1 upregulation during muscle atrophy and reduces muscle loss. Many signaling pathways and molecules involved in skeletal muscle catabolism and anabolism have been reported in various literature in recent years. Whether these pathways and molecules play comparable roles in the coexistence of COPD and sarcopenia requires further investigation. A special oral nutrition supplement rich in whey and casein proteins that had high levels of branched amino acids, vitamin D, and ursolic acid, was fed to a muscle atrophy mouse model induced by restricted calorie intake, and the catabolic and anabolic signaling pathways in the muscles were investigated. Notably, these pathways were attenuated (56). However, whether the same mechanism is present in patients with coexisting COPD and sarcopenia remains to be investigated.

### 2.5. Unhealthy lifestyles

#### 2.5.1. Smoking

Smoking is the most important risk factor for the development of COPD as it induces not only COPD but also skeletal muscle dysfunction, and the mechanisms may have commonalities with those of the underlying coexisting COPD and sarcopenia. Carbon monoxide in cigarette smoke can reduce the oxygen-carrying capacity of blood and induce a left shift in the hemoglobin dissociation curve, binding myoglobin and impeding the diffusion of intracellular oxygen, which eventually leads to a reduction in the oxygen supply to mitochondria in myocytes and an impaired ability of mitochondria to utilize oxygen (57). Chronic cigarette smoke exposure can degenerate the neuromuscular junction and also activate the MAPK and NF-κB signaling pathways, resulting in increased myosin hydrolysis (32). Oxidative stress in skeletal muscle can be increased by the free radicals in cigarette smoke (58, 59). Cigarette smoke also contains various harmful substances that interfere with the mitochondrial respiratory chain and prevent mitochondria from producing ATP, impairing skeletal muscle function (60). Mice exposed to cigarette smoke have reduced Ca^2+^ transients in skeletal muscle fibers independent of Ca^2+^ release from the SR (61). The inhibition of the lymphotoxin β- receptor signaling pathway induced lung regeneration, attenuated airway fibrosis, and alleviated systemic muscle atrophy in mice exposed to cigarette smoke (62). *In vitro* studies have shown a decrease in myosin in cigarette smoke-exposed myocytes, accompanied by the elevated expression of MAFBx, MuRF-1, and ubiquitin-specific proteases (63–65). One study found that cigarette smoke extract induced ferroptosis via the HIF2α pathway in C2C12 myotubes (66). Furthermore, studies have demonstrated that cigarette smoke can inactivate Akt, thus inhibiting myocyte protein synthesis, which causes muscle atrophy. Altered protein number and improvement in phosphorylation status have been shown after 60 days of smoking cessation in mice (67). An animal study showed that myocyte mitochondrial dysfunction, loss of limb muscle mass, and diaphragm atrophy caused by cigarette smoke were reversed by short-term smoking cessation (68). Given the role of smoking cessation in stopping or slowing the progression of COPD, various studies have suggested that smoking cessation may have beneficial effects on muscle function in patients with COPD.

#### 2.5.2. Alcohol

Chronic alcohol abuse is a risk factor for COPD. The metabolites of alcohol, acetaldehyde, and ROS can cause oxidative stress through mechanisms related to both metabolic pathways of alcohol: the oxidative pathway catalyzed by hepatic alcohol dehydrogenase and the metabolic pathway via cytochrome P450 2E1 (CYP2E1) (69). Chronic alcohol intake often leads to alcoholic myopathy via impaired mitochondrial function and self-regeneration in skeletal muscle cells, increased inflammatory and fibrotic markers, and changes in pathways regulating skeletal muscle anabolism and catabolism (70–72). IGF-1 level was significantly decreased both in the plasma and muscles. Moreover, the mTOR pathway was directly affected by alcohol (73). An alternative pathway controlling muscle mass is activated mainly by TGF-β, activin, and MSTN of the Smad family. Alcohol may also interact with the ubiquitin–proteasome pathway, affecting protein degradation (74). Rats chronically ingesting alcohol showed plantaris muscle atrophy, which is mediated by the overexpression of both MuRF-1 and MAFbx (75). The aforementioned pathways are similar to those involved in COPD-induced muscle atrophy. Although there is evidence that patients with COPD seemingly have a higher alcohol intake (76), the study population was from regions at high latitudes, and the findings of this study cannot be extrapolated with certainty to patients globally. Even in young individuals, repeated occasional high alcohol intake can lead to increased levels of protein nitration, elevated levels of oxidative damage to nucleic acids, smaller muscle fiber size, and increased profibrotic factors, all of which eventually lead to decreased muscle strength (77). Therefore, for patients with coexisting COPD and sarcopenia, it is advisable to refrain from alcohol entirely. The mechanisms of muscle atrophy and decreased muscle function due to alcoholic myopathy have been well investigated; however, the mechanism of the negative effects on muscle caused by the conjunction of COPD and alcohol remains unclear. Hence, the alcohol consumption habits of patients with COPD and the effects of alcohol on their skeletal muscles need to be elucidated in controlled clinical studies with large sample sizes.

#### 2.5.3. Reduced physical activity

People with COPD often have breathing difficulties due to progressive irreversible airflow restriction, which may reduce their daily physical activity. Prolonged reduction in physical activity may lead to skeletal muscle waste, which may cause several adaptive changes, including reduced type I fibers and oxidase capacity, muscle fiber atrophy, and decreased muscle capillaries (78). These changes further lead to a decrease in muscle strength and endurance. Diseases caused by inflammation and acute hypoxia, such as COVID-19 infection, significantly reduce the activity level of patients. Based on the pathogenesis of muscle wasting, inflammatory factors are released in abundance during the initiation of the disease which can further exacerbate muscle atrophy (79). Since COPD is an inflammatory disease, it is speculated that its inflammatory properties may accelerate the rate of muscle atrophy, further reducing the exercise endurance of patients with COPD and, consequently, leading to a vicious cycle of disease progression.

Muscle protein breakdown (MPB) and muscle protein synthesis (MPS) occur in skeletal muscle in synchronous succession, and their dynamic balance together determines the quality of skeletal muscle (80). One study comprising patients with COPD, who may have had reduced physical activity status (non-sarcopenic COPD, *n* = 53; sarcopenic COPD, *n* = 39), and a healthy non-sarcopenic control group (*n* = 13) evaluated a comprehensive set of muscle protein turnover molecular regulators by performing muscle biopsy of the lateralis femoris muscle, demonstrating increased protein degradation and synthetic signaling in skeletal muscle in patients with COPD (81). This study suggests that the increased myogenic signaling observed indicates enhanced muscle repair and remodeling in these patients. Several studies have shown that rather than increase in MPB, MPS is the main determinant of disuse muscular atrophy in humans (82–84). However, some of these findings are based on short-term (≤3 weeks) muscle disuse in healthy individuals; in comparison, muscle disuse in patients with coexisting COPD and sarcopenia is long-lasting and more complicated, and further research is needed to determine whether MPS is the main determinant in those patients. An increase or decrease in MPB and MPS has different effects in patients with coexisting COPD and sarcopenia than in those with disuse muscular atrophy alone. Studies have also shown significant differences in quadricep muscle endurance between patients with COPD and healthy subjects, even when their physical activity levels were similar (85). Accordingly, we speculate that reduced physical activity can only partially explain the pathogenesis of COPD combined with sarcopenia and that more pathophysiological factors are involved. Combining aerobic and resistance training with pulmonary rehabilitation in patients with COPD improved their muscle endurance (86). Moreover, they improved muscle strength and gait speed in elderly patients with sarcopenia, but did not shorten the time of the 5 times sit-to-stand test (87). The development of individualized rehabilitation exercise strategies for patients with coexisting COPD and sarcopenia may be beneficial for their functional maintenance.

### 2.6. Other possible causes

Experimental studies have found that the activated ERK1/2 subfamily of the MAPK signaling pathway was involved in the slow-to-fast muscle fiber shift (88). Thus, similar mechanisms may cause the muscle fiber shift in the lower limbs of patients with COPD.

The Klotho gene, detected in 1997, was first thought to be an aging-suppressor gene (89). Homozygous mutant Klotho (KL-/-) mice display a variety of premature aging phenotypes, including emphysema and sarcopenia, and their lifespan is considerably shortened (90). Studies have demonstrated that Klotho deficiency substantially affects muscle strength, exercise endurance, and physical activity in mice (89, 91). The Klotho protein consists of α-klotho and β-klotho. β-klotho is a major component of the endocrine fibroblast growth factor (FGF) receptor complex. Animal studies showed that the exogenous FGF 19 administration improved muscle mass and grip strength, and increased the transverse diameter of their myotubes (92); serum FGF 21 levels are positively correlated with aging-induced sarcopenia (93). In a study of older adults (most of whom had sarcopenia or probable sarcopenia), FGF 19 levels were negatively correlated with muscle fiber length (94). However, no studies have investigated the role of nutritional supplementation of the Klotho protein expression to improve muscle size or physical performance in patients with COPD and sarcopenia. The Klotho gene is a potential pharmacological therapeutic target.

Recently, researchers have identified irisin as a hormone secreted by skeletal myocytes (95), which is reportedly associated with exercise capacity and skeletal muscle dysfunction in patients with COPD (96). Its underlying mechanisms may involve (i) its ability to increase the expression of the peroxisome proliferator-activated receptor-γ coactivator-1-α (*PGC-1*α) gene, which in turn increases the mitochondrial content of myocytes and improves mitochondrial function (97); (ii) the antagonism of irisin with MSTN via the PGC-1α-FNDC5/Irisin pathway (98). Since the above mechanisms may be limited to sarcopenia alone, it is still necessary to conduct further in-depth studies to determine whether the above mechanisms play a similar role in patients with coexisting COPD and sarcopenia.

## 3. Role of nutrition among patients with coexisting COPD and sarcopenia

### 3.1. Macronutrients

#### 3.1.1. Protein and amino acids

It has been proved that body weight, muscle strength, exercise capability, and quality of life can be improved by a combination of nutritional supplements, exercise/pulmonary rehabilitation, and medical treatment in patients with respiratory disease (99, 100). Importantly, these studies have consistently reported an increase in fat-free mass (FFM).

Protein intake is particularly associated with muscle mass maintenance. Elderly patients with sarcopenia, especially those with COPD, have reduced protein synthesis and a high catabolic state (101, 102) that may further increase their protein requirements (103, 104). Therefore, adequate protein intake is critical for individuals participating in an exercise program, whether in a healthy or diseased state, for it can increase protein metabolism and promote muscle repair. In addition, the Health ABC study showed that among older adults, higher protein intake reduced lean muscle mass loss over time, strongly supporting the idea that older adults with sarcopenia should have an adequate protein intake (105). Consensus on the optimal level of protein ingestion for sarcopenia intervention is still undetermined, with recent medical suggestions substantially varying between 1.2 and 2.0 g/kg (106). Essential amino acids (EAA) such as leucine and its metabolite beta-hydroxy-beta-methyl butyrate (HMB) appear to be particularly important for increasing FFM, muscle mass, and strength when protein is supplemented (107).

Amino acids are essential components of proteins, and multiple studies have reported decreased plasma amino acid levels in patients with COPD having lower body weight or reduced muscle mass (108, 109). Leucine is three times more potent than other essential amino acids in stimulating anabolic signals in skeletal muscles (110). Therefore, the potential role of leucine in the treatment of coexisting COPD and sarcopenia has aroused renewed interest. The PROVIDE study, which comprised elderly individuals with sarcopenia, investigated the effectiveness of a nutritional supplement rich in vitamin D and leucine compared to an isocaloric control and showed that the former was more effective in improving physical function (5 times sit-to-stand test) and appendicular muscle mass (111). HMB, a metabolite of active leucine, prevents the loss of FFM during muscle disuse (112). In a cohort study comprising hospitalized elderly patients, HMB yielded improved outcomes in the treatment of malnutrition (113). The effect of nutritional support with HMB as the main component in improving muscle mass is independent of exercise training; hence, HMB is a possible nutritional supplement for patients with limited exercise capacity or bedridden patients. Unsurprisingly, nutritional supplements combined with exercise improved the performance of patients with coexisting COPD and sarcopenia. A clinical study included 81 patients with COPD and low muscle mass who participated in an outpatient pulmonary rehabilitation program consisting primarily of supervised high-intensity exercise training with randomized oral nutritional supplements rich in leucine, vitamin D, and omega-3 fatty acids or placebo. The results showed that specific nutritional supplementation alongside high-intensity exercise training had additional benefits on overall nutritional status, inspiratory muscle strength, and physical activity among patients with COPD, moderate airflow obstruction, and low muscle mass (114).

Involuntary weight loss is commonly seen in patients with COPD (103, 115). In contrast to primary sarcopenia, malnutrition and increased energy requirements are associated with COPD owing to high metabolism and increased whole-body protein turnover (109, 116). Elderly individuals with sarcopenia, especially those with COPD, who have reduced anabolic response to protein intake and are in a hypermetabolic state may need more protein supplements than healthy older adults (103, 108). Therefore, adequate protein and amino acid supplementation together with exercise programs play a key role in the rehabilitation of elderly patients with coexisting COPD and sarcopenia.

#### 3.1.2. Carbohydrates and fats

Increasing calorie intake in patients with COPD can significantly increase body weight and muscle strength through nutritional supplementation together with nutritional advice, improving the quality of life of patients and, in turn, potentially reducing mortality rates (24). The hypothesis that high-fat supplements may be more beneficial than high-carbohydrate supplements in increasing calorie intake in patients with COPD has been proposed in several studies (117–119).

Some fatty acids, including polyunsaturated fatty acids (PUFA), especially omega-3 polyunsaturated fatty acids, have been demonstrated to play a role in various inflammatory and metabolic pathways (120–122). Moreover, systemic inflammation is related to muscle atrophy and decreased muscle function in patients with COPD (78). Accordingly, several studies have inferred that PUFA may be involved in the pathogenesis of muscle atrophy in patients with COPD (123–125).

Multiple clinical and experimental studies have suggested that PUFA may improve response to pulmonary rehabilitation (126). In 32 clinically stable patients with malnutrition combined with moderate to severe COPD, body weight, muscle mass, and strength, as well as functional performance, increased with 1.2 g/d n-3 PUFAs combined with low-intensity exercise for 12 weeks (125). However, a controlled trial in the Netherlands included 80 patients with COPD undergoing pulmonary rehabilitation and randomly divided them into the PUFA and placebo groups, which were administered 9 g of PUFA and isocaloric placebo per day, respectively. Compared with the placebo group, the PUFA group had more peak loads of the incremental exercise test; however, in terms of weight, FFM, and muscle strength, the two groups both showed a similar degree of increase. Therefore, the role of n-3 fatty acids in patients undergoing rehabilitation needs further study (123). Moreover, an 8-week clinical cohort study comprising 32 patients with moderate-to-severe COPD that were provided supplements of n-3 PUFA (1,200 mg ALA, 700 mg EPA, and 340 mg DHA) achieved reversed muscle atrophy and improved muscle function compared with placebo. However, after rehabilitation or PUFA intervention, the systemic inflammation markers (CRP, IL-6, and TNF-α) did not change (123). Nevertheless, a clinical study revealed a correlation between serum inflammatory markers in clinically stable patients with COPD and their dietary intake of omega-3 and omega-6 fatty acids; specifically, individuals with high levels of alpha-linolenic acid intake had lower serum TNF-α concentrations and individuals with high intakes of arachidonic acid had higher levels of IL-6 and CRP (127). These contradictory results may be related to differences in the dose of PUFA, the number of people included, or the duration of observation. It has also been proposed that the mitochondrial membrane composition and respiratory dynamics in human skeletal muscle can be altered by supplementation with omega-3 fatty acids, thereby improving exercise performance to some extent by improving metabolic efficiency during exercise (128). Regardless of the mechanism underlying COPD and sarcopenia, n-3 PUFA supplements are recommended for the nutritional management of chronic diseases owing to their positive effects on muscle health (129).

### 3.2. Micronutrients

#### 3.2.1. Vitamins

The role of vitamins has received increasing attention for the treatment of coexisting COPD and sarcopenia.

Patients with COPD frequently suffer from vitamin D deficiency due to reduced activity, insufficient sunlight, and decreased synthesis of vitamin D in the elderly owing to changes in skin status and decreased organ function (103, 130). Vitamin D is a fat-soluble steroid hormone that has a major function in regulating calcium and phosphorus homeostasis and in maintaining muscle and bone health. The active form of vitamin D, 1,25-dihydroxyvitamin D3, controls the expression of various genes through binding to the vitamin D receptor (VDR) and produces physiological effects via genomic and non-genomic pathways (131). VDR is expressed in the skeletal muscle and induces muscle protein synthesis. The binding of vitamin D to VDR stimulates the absorption of inorganic phosphate within cells, which is used to produce energy-rich phosphate compounds that are essential for maintaining muscle contractions. Vitamin D supplementation in patients with COPD can exert complex anti-inflammatory effects, decreases excessive ROS production, enhances VDR gene expression and protein levels, and prevents muscle wasting (132). One study showed that patients with COPD who had higher levels of vitamin D exhibited superior muscle strength and quality of life when compared to those with lower vitamin D levels (133), confirming that vitamin D has an important effect on skeletal muscle function. Vitamin D influences muscle metabolism, and its supplementation has been demonstrated to reduce the rate of COPD exacerbation, which could indirectly, potentially decelerate sarcopenia progression in patients with COPD (134). Moreover, vitamin D regulates FOXO3 and Notch signaling, promotes myoblast self-renewal, maintains the satellite stem cell pool, and benefits regeneration and repair after muscle injury (135). However, there are conflicting reports regarding the effectiveness of vitamin D supplementation as a therapy for COPD. A single study demonstrated that the frequency of moderate/severe COPD exacerbations was significantly and safely reduced by vitamin D supplementation, but only in patients with baseline 25-hydroxyvitamin D levels below 25 nmol/L (134). A study on the effects of vitamin D supplementation in patients with COPD who were vitamin D deficient revealed an advantageous impact of taking vitamin D supplements on muscle health (136). Another study showed that supplementing with vitamin D_3_ did not enhance the effects of resistance training in elderly individuals (137). Based on these contradictory findings, large cross-sectional and prospective studies are needed to confirm whether vitamin D affects skeletal muscle dysfunction in patients with COPD.

Individuals without sarcopenia consume more vitamin K in their diet compared to those with sarcopenia (138). Vitamin K may play a favorable role through various mechanisms. Vitamin K can enhance muscle function by promoting vascular smooth muscle differentiation, improving arterial function and muscle perfusion, and serving as an electron carrier in skeletal muscle mitochondria. There is evidence to suggest that low concentration of vitamin K is involved in the development of COPD (139), and an increased risk of emphysema is associated with a low intake of vitamin K (140). However, further research to establish a direct causal relationship between vitamin K intake and COPD is warranted.

#### 3.2.2. Minerals

Several minerals have been confirmed to be associated with skeletal muscle mass. Among European white people, high iron levels predicted by genetic phenotypes are positively correlated with sarcopenia, and serum calcium levels have a potential negative correlation with sarcopenia (141). Iron accumulation-induced skeletal muscle atrophy is probably related to mitochondrial function, oxidative damage, and the ubiquitin-proteasome pathway (142–145). However, contradictory results have been found with iron levels and muscle function. Notably, a recent study found that iron-deficient patients with severe COPD had low serum ferritin levels that correlated significantly with walking distance as measured by the six-minute walking test, suggesting a decrease in muscle function (146).

Calcium is necessary for healthy muscle and nerve activity, while selenium has antioxidant properties. Observational studies have shown that serum calcium (147) and selenium (148) intakes are significantly associated with muscle mass. Meanwhile, the calcium sensitizer levosimendan may enhance force-generating capacity, and improve the neuromechanical efficiency and contractile function of diaphragm fibers by increasing calcium sensitivity (149, 150). However, no studies have been carried out on the effect of this medication on limb muscle contractility in patients with coexisting COPD and sarcopenia. A previous study has shown that calcium release and the maximal efflux rate from the SR could be increased by selenium supplementation, which could also improve the *in vivo* and *in vitro* skeletal muscle performance (151). Another study showed that when the combined dietary intake of vitamins A, E, B_6_, B_12_, folate, selenium, and zinc was reduced in aged mice, lower oxidative capacity, muscle mitochondrial capacity, and muscle fiber atrophy were observed (152). Additionally, the positive effects of selenium on skeletal muscles seem to be independent of testosterone levels (153, 154).

Zinc slows down oxidative processes in the body and also inhibits aromatase and 5α-reductase, which are necessary for testosterone metabolism (155). The use of antioxidant supplements containing zinc gluconate and selenium in combination with pulmonary rehabilitation improves quadriceps strength and increases the proportion of type I fibers in patients with COPD (156).

Magnesium is considered a predictor of disease severity in patients with COPD and can reduce systemic inflammation and pro-inflammatory cytokine levels (157). The concentration of ionized magnesium in polymorphonuclear cells was significantly lower in patients with COPD than those of healthy non-smokers. Moreover, the total Ca/total Mg ratios in the plasma and polymorphonuclear cells of patients with COPD were significantly greater than those of healthy non-smokers and smokers (158). A randomized controlled trial demonstrated increased whole-body fat-free mass, FFMI, and muscle strength in patients with moderate-to-severe COPD who consumed whey beverages fortified with magnesium and vitamin C (159). Other studies have also confirmed that higher magnesium intake is significantly and positively associated with appendicular lean mass (160). Moreover, serum magnesium concentration is independently correlated with muscle strength (161).

Clinical studies have reported reduced serum phosphorus levels in patients with COPD (162–164). Fructose 1,6-diphosphate was administered to malnourished patients with COPD in one study and it was found to increase respiratory muscle strength; however, the effect of the medication on skeletal muscle strength of the limbs has not yet been studied (165). Other micronutrients, including copper, manganese, molybdenum, and lithium, have not yet been studied for their roles in patients with coexisting COPD and sarcopenia. Minerals generally exert positive effects on the muscles through indirect approaches. Further studies are required to formulate an appropriate mineral supplementation regimen for patients with coexisting COPD and sarcopenia.

### 3.3. Drugs affecting muscle metabolism

A combination of exercise training and nutritional supplementation may not always be beneficial for patients with coexisting COPD and sarcopenia. Therefore, pharmacological approaches could serve as an important third choice in diversified treatment programs for these patients.

Testosterone and anabolic steroids are known to increase muscle mass and reduce fat mass in young and healthy adults. Notably, they exert similar effects in patients with sarcopenia (166). Testosterone use, alone or in conjunction with exercise, has been reported to be related to reduced hospitalization, increased FFM, and muscle strength in patients with COPD, but without strengthened exercise ability (167–170). Although nandrolone decanoate (ND), the synthetic form of testosterone, reportedly increased exercise capacity in patients taking glucocorticoid medication, intramuscular injection of anabolic steroids in the form of ND provided during pulmonary rehabilitation did not improve exercise capacity, but only showed an increase in FFM. However, testosterone should be used with caution owing to its significant side effects including cardiovascular events, hepatotoxic, renal, gastrointestinal, and endocrine effects (168). However, a recent study on aged rats showed that ND had no significant counteracting effect on muscle regeneration decline associated with aging (171).

Other hormones that have been shown to benefit patients with coexisting COPD and sarcopenia include growth hormone (GH) and ghrelin. GH supplementation increases body weight, muscle mass, and respiratory muscle strength in patients with COPD via several pathways (172, 173). Unfortunately, its clinical application is limited by its negative side effects, which are similar to those of testosterone (174). Effects of ghrelin on appetite and feeding have also been demonstrated. For patients with COPD or coexisting COPD and sarcopenia who have decreased appetite and inadequate caloric intake, preventing weight loss by appetite stimulation may be an appropriate approach (174). Improvements in exercise capacity following ghrelin supplementation in patients with COPD have been reported (166, 175), even though not all studies showed its positive impact on functionality or muscle strength (166).

Myostatin is a potent negative regulator of muscle mass and is elevated in patients with COPD. Improving muscle growth via the inhibition of MSTN or antagonizing its receptor (activin IIR) may be feasible (166, 174). Bimagrumab, an activin IIR antagonist, has been shown to improve muscle mass, strength, and mobility in elderly patients with sarcopenia living in the community (176). However, it did not improve functional capacity in patients with COPD and it only increased the muscle mass (177). The aforementioned study suggests that improving muscle mass using anabolic drugs alone may not be sufficient to improve physical performance; however, some COPD-specific factors may impact physical performance by affecting muscle mass.

Current research on the treatment of patients with coexisting COPD and sarcopenia is still in its preliminary stage. Although many studies have demonstrated the positive effects of these drugs, their clinical application remains limited due to their side effects. In particular, glucocorticoids can damage multiple systems. Therefore, it is crucial for establishing further studies on the mechanisms and safety of potential pharmacological treatment of coexisting COPD and sarcopenia.

## 4. Summary and conclusion

Current studies suggest that the pathogenesis of COPD and sarcopenia is similar to that of other muscle loss-inducing diseases, including myotonic dystrophy and alcoholic myopathy. Although many studies have explored various mechanisms in the pathogenesis of sarcopenia alone, there are few studies investigating the mechanisms of COPD and sarcopenia. Furthermore, there are few longitudinal studies on the pathogenesis of COPD and sarcopenia. Many studies have focused on improving muscle loss in these patients through nutritional support and have shown promising results, despite few studies reporting contradictory results. A controlled trial in the Netherlands found that nutritional interventions (a mixture of protein, carbohydrates, fats, and micronutrients rich in leucine, Omega-3 PUFA, and vitamin D) in patients with COPD and muscular atrophy may not improve the long-term effectiveness of exercise training (178). However, the underlying mechanisms are yet to be clarified. Standardized experiments regarding the management of nutritional support for patients with COPD and sarcopenia, as well as exploratory studies on the intrinsic mechanisms of nutritional supplementation in these patients are warranted.

## Author contributions

YN and LP contributed to the conception and framework of the manuscript. YN, LP, YZ, ZD, TY, and PZ searched and analyzed the literatures. YN, LP, YZ, ZD, and PZ wrote the sections of the manuscript. YZ made the table. FZ and QC helped perform the analysis with constructive discussions. All authors contributed to manuscript revision, read, and approved the submitted version.

## References

[1] The Lancet. *The lancet 2019 global burden of disease.* (2020). Available online at: https://www.thelancet.com/gbd/summaries (accessed March 29, 2023).

[2] Tsekoura M Tsepis E Billis E Gliatis J. Sarcopenia in patients with chronic obstructive pulmonary disease: a study of prevalence and associated factors in western Greek population. *Lung India.* (2020) 37:479–84. 10.4103/lungindia.lungindia_143_20 33154208 PMC7879857

[3] Jones S Maddocks M Kon S Canavan J Nolan C Clark A Sarcopenia in COPD: prevalence, clinical correlates and response to pulmonary rehabilitation. *Thorax.* (2015) 70:213–8. 10.1136/thoraxjnl-2014-206440 25561517

[4] Kaluźniak-Szymanowska A Krzymińska-Siemaszko R Deskur-Śmielecka E Lewandowicz M Kaczmarek B Wieczorowska-Tobis K. Malnutrition, sarcopenia, and malnutrition-sarcopenia syndrome in older adults with COPD. *Nutrients.* (2021) 14:44. 10.3390/nu14010044 35010919 PMC8746722

[5] Perrot L Greil A Boirie Y Farigon N Mulliez A Costes F Prevalence of sarcopenia and malnutrition during acute exacerbation of COPD and after 6 months recovery. *Eur J Clin Nutr.* (2020) 74:1556–64. 10.1038/s41430-020-0623-6 32296123

[6] Chung J Hwang H Han C Son B Kim D Park M. Association between sarcopenia and metabolic syndrome in chronic obstructive pulmonary disease: the Korea National Health and Nutrition Examination Survey (KNHANES) from 2008 to 2011. *COPD.* (2015) 12:82–9. 10.3109/15412555.2014.908835 24914701

[7] Gologanu D Ionita D Gartonea T Stanescu C Bogdan M. Body composition in patients with chronic obstructive pulmonary disease. *Maedica.* (2014) 9:25–32. 25553122 PMC4268286

[8] Trajanoska K Schoufour J Darweesh S Benz E Medina-Gomez C Alferink L Sarcopenia and its clinical correlates in the general population: the Rotterdam study. *J Bone Miner Res.* (2018) 33:1209–18. 10.1002/jbmr.3416 29502340

[9] Cebron Lipovec N Schols A van den Borst B Beijers R Kosten T Omersa D Sarcopenia in advanced COPD affects cardiometabolic risk reduction by short-term high-intensity pulmonary rehabilitation. *J Am Med Direct Assoc.* (2016) 17:814–20. 10.1016/j.jamda.2016.05.002 27321867

[10] Limpawattana P Inthasuwan P Putraveephong S Boonsawat W Theerakulpisut D Sawanyawisuth K. Sarcopenia in chronic obstructive pulmonary disease: a study of prevalence and associated factors in the Southeast Asian population. *Chronic Respir Dis.* (2018) 15:250–7. 10.1177/1479972317743759 29186972 PMC6100162

[11] Demircioğlu H Cihan F Kutlu R Yosunkaya Ş Zamani A. Frequency of sarcopenia and associated outcomes in patients with chronic obstructive pulmonary disease. *Turk J Med Sci.* (2020) 50:1270–9. 10.3906/sag-1909-36 32421282 PMC7491298

[12] Persson H Sioutas A Kentson M Jacobson P Lundberg P Dahlqvist Leinhard O Skeletal myosteatosis is associated with systemic inflammation and a loss of muscle bioenergetics in stable COPD. *J Inflammation Res.* (2022) 15:4367–84. 10.2147/jir.S366204 35937916 PMC9355337

[13] Budweiser S Meyer K Jörres R Heinemann F Wild P Pfeifer M. Nutritional depletion and its relationship to respiratory impairment in patients with chronic respiratory failure due to COPD or restrictive thoracic diseases. *Eur J Clin Nutr.* (2008) 62:436–43. 10.1038/sj.ejcn.1602708 17342162

[14] Schols A Broekhuizen R Weling-Scheepers C Wouters E. Body composition and mortality in chronic obstructive pulmonary disease. *Am J Clin Nutr.* (2005) 82:53–9. 10.1093/ajcn.82.1.53 16002800

[15] Vestbo J Prescott E Almdal T Dahl M Nordestgaard B Andersen T Body mass, fat-free body mass, and prognosis in patients with chronic obstructive pulmonary disease from a random population sample: findings from the Copenhagen city heart study. *Am J Respir Crit Care Med.* (2006) 173:79–83. 10.1164/rccm.200506-969OC 16368793

[16] Sepúlveda-Loyola W Osadnik C Phu S Morita A Duque G Probst V. Diagnosis, prevalence, and clinical impact of sarcopenia in COPD: a systematic review and meta-analysis. *J Cachexia Sarcopenia Muscle.* (2020) 11:1164–76. 10.1002/jcsm.12600 32862514 PMC7567149

[17] Kim S Yoon H Rhee C Jung H Lee H Jo Y. Hand grip strength and likelihood of moderate-to-severe airflow limitation in the general population. *Int J Chronic Obstr Pulm Dis.* (2022) 17:1237–45. 10.2147/copd.S364351 35642183 PMC9148604

[18] Kitamura A Seino S Abe T Nofuji Y Yokoyama Y Amano H Sarcopenia: prevalence, associated factors, and the risk of mortality and disability in Japanese older adults. *J Cachexia Sarcopenia Muscle.* (2021) 12:30–8. 10.1002/jcsm.12651 33241660 PMC7890144

[19] Feliciano E Kroenke C Meyerhardt J Prado C Bradshaw P Kwan M Association of systemic inflammation and sarcopenia with survival in nonmetastatic colorectal cancer: results from the C scans study. *JAMA Oncol.* (2017) 3:e172319. 10.1001/jamaoncol.2017.2319 28796857 PMC5824285

[20] Carbone S Kirkman D Garten R Rodriguez-Miguelez P Artero E Lee D Muscular strength and cardiovascular disease: an updated state-of-the-art narrative review. *J Cardiopulm Rehabil Prev.* (2020) 40:302–9. 10.1097/HCR.0000000000000525 32796492

[21] Okazaki T Ebihara S Mori T Izumi S Ebihara T. Association between sarcopenia and pneumonia in older people. *Geriatr Gerontol Int.* (2020) 20:7–13. 10.1111/ggi.13839 31808265

[22] van Bakel S Gosker H Langen R Schols A. Towards personalized management of sarcopenia in COPD. *Int J Chronic Obstr Pulm Dis.* (2021) 16:25–40. 10.2147/copd.S280540 33442246 PMC7800429

[23] World Health Organization [WHO]. *World report on ageing and health.* (2015). Available online at: https://www.who.int/publications/i/item/9789241565042 (accessed July 15, 2023).

[24] Hsieh M Yang T Tsai Y. Nutritional supplementation in patients with chronic obstructive pulmonary disease. *J Formosan Med Assoc.* (2016) 115:595–601. 10.1016/j.jfma.2015.10.008 26822811

[25] Späte U Schulze P. Proinflammatory cytokines and skeletal muscle. *Curr Opin Clin Nutr Metab Care.* (2004) 7:265–9. 10.1097/00075197-200405000-00005 15075917

[26] Sharma B Dabur R. Role of pro-inflammatory cytokines in regulation of skeletal muscle metabolism: a systematic review. *Curr Med Chem.* (2020) 27:2161–88. 10.2174/0929867326666181129095309 30488792

[27] Rong Y Bian A Hu H Ma Y Zhou X. Study on relationship between elderly sarcopenia and inflammatory cytokine Il-6, anti-inflammatory cytokine Il-10. *BMC Geriatr.* (2018) 18:308. 10.1186/s12877-018-1007-9 30541467 PMC6292155

[28] Zhang Y Zuo H Tian D Ouyang X Wang X. Correlation between peripheral skeletal muscle functions and the stable phase of COPD in older patients. *Eur Rev Med Pharmacol Sci.* (2018) 22:5317–26. 10.26355/eurrev_201808_15732 30178857

[29] Cruz B Oliveira A Gomes-Marcondes MCC. L-leucine dietary supplementation modulates muscle protein degradation and increases pro-inflammatory cytokines in tumour-bearing rats. *Cytokine.* (2017) 96:253–60. 10.1016/j.cyto.2017.04.019 28494385

[30] Doucet M Russell A Leger B Debigare R Joanisse D Caron M Muscle atrophy and hypertrophy signaling in patients with chronic obstructive pulmonary disease. *Am J Respir Crit Care Med.* (2007) 176:261–9. 10.1164/rccm.200605-704OC 17478621

[31] Kim Y Kim C Joe Y Chung H Ha T Yu R. Quercetin reduces tumor necrosis factor alpha-induced muscle atrophy by upregulation of heme oxygenase-1. *J Med Food.* (2018) 21:551–9. 10.1089/jmf.2017.4108 29569982

[32] Kaisari S Rom O Aizenbud D Reznick A. Involvement of Nf-Kappab and muscle specific E3 ubiquitin ligase Murf1 in cigarette smoke-induced catabolism in C2 myotubes. *Adv Exp Med Biol.* (2013) 788:7–17. 10.1007/978-94-007-6627-3_2 23835952

[33] Chen S Gerken E Zhang Y Zhan M Mohan R Li A Role of Tnf-alpha signaling in regeneration of cardiotoxin-injured muscle. *Am J Physiol Cell Physiol.* (2005) 289:C1179–87. 10.1152/ajpcell.00062.2005 16079187 PMC3099530

[34] Bagheri A Hashemi R Soltani S Heshmat R Dorosty Motlagh A Larijani B The relationship between food-based pro-inflammatory diet and sarcopenia: findings from a cross-sectional study in Iranian elderly people. *Front Med.* (2021) 8:649907. 10.3389/fmed.2021.649907 34041251 PMC8141626

[35] Gojanovic M Holloway-Kew K Hyde N Mohebbi M Shivappa N Hebert J The dietary inflammatory index is associated with low muscle mass and low muscle function in older Australians. *Nutrients.* (2021) 13:1166. 10.3390/nu13041166 33916033 PMC8065722

[36] Su Y Yeung S Chen Y Leung J Kwok T. The associations of dietary inflammatory potential with musculoskeletal health in Chinese community-dwelling older people: the Mr. Os and Ms. Os (Hong Kong) cohort study. *J Bone Miner Res.* (2022) 37:1179–87. 10.1002/jbmr.4556 35416312 PMC9177744

[37] Ticinesi A Meschi T Lauretani F Felis G Franchi F Pedrolli C Nutrition and inflammation in older individuals: focus on vitamin D, N-3 polyunsaturated fatty acids and whey proteins. *Nutrients.* (2016) 8:186. 10.3390/nu8040186 27043616 PMC4848655

[38] Jin M Bok M Rho H Chon J Lim HA. Pro-inflammatory diet increases the risk of sarcopenia components and inflammatory biomarkers in postmenopausal women. *Nutr Res.* (2022) 107:195–205. 10.1016/j.nutres.2022.09.008 36323193

[39] Qaisar R Ustrana S Muhammad T Shah I. Sarcopenia in pulmonary diseases is associated with elevated sarcoplasmic reticulum stress and myonuclear disorganization. *Histochem Cell Biol.* (2022) 157:93–105. 10.1007/s00418-021-02043-3 34665327

[40] Green H Burnett M Duhamel T D’Arsigny C O’Donnell D Webb K Abnormal sarcoplasmic reticulum Ca^2+^-sequestering properties in skeletal muscle in chronic obstructive pulmonary disease. *Am J Physiol Cell Physiol.* (2008) 295:C350–7. 10.1152/ajpcell.00224.2008 18508908

[41] Ju C Chen M Zhang J Lin Z Chen R. Higher plasma myostatin levels in cor pulmonale secondary to chronic obstructive pulmonary disease. *PLoS One.* (2016) 11:e0150838. 10.1371/journal.pone.0150838 26998756 PMC4801210

[42] Plant P Brooks D Faughnan M Bayley T Bain J Singer L Cellular markers of muscle atrophy in chronic obstructive pulmonary disease. *Am J Respir Cell Mol Biol.* (2010) 42:461–71. 10.1165/rcmb.2008-0382OC 19520920

[43] Hayot M Rodriguez J Vernus B Carnac G Jean E Allen D Myostatin up-regulation is associated with the skeletal muscle response to hypoxic stimuli. *Mol Cell Endocrinol.* (2011) 332:38–47. 10.1016/j.mce.2010.09.008 20884321

[44] Srivastava S Rathor R Singh S Suryakumar G. Insight into the role of myokines and myogenic regulatory factors under hypobaric hypoxia induced skeletal muscle loss. *Biomarkers.* (2022) 27:753–63. 10.1080/1354750X.2022.2112290 35946424

[45] Reed S Sandesara P Senf S Judge A. Inhibition of foxo transcriptional activity prevents muscle fiber atrophy during cachexia and induces hypertrophy. *FASEB J.* (2012) 26:987–1000. 10.1096/fj.11-189977 22102632 PMC3289501

[46] Parandak K Arazi H Khoshkhahesh F Nakhostin-Roohi B. The effect of two-week L-carnitine supplementation on exercise -induced oxidative stress and muscle damage. *Asian J Sports Med.* (2014) 5:123–8. 25834706 PMC4374610

[47] Haji G Wiegman C Michaeloudes C Patel M Curtis K Bhavsar P Mitochondrial dysfunction in airways and quadriceps muscle of patients with chronic obstructive pulmonary disease. *Respir Res.* (2020) 21:262. 10.1186/s12931-020-01527-5 33046036 PMC7552476

[48] Leermakers P Schols A Kneppers A Kelders M de Theije C Lainscak M Molecular signalling towards mitochondrial breakdown is enhanced in skeletal muscle of patients with chronic obstructive pulmonary disease (COPD). *Sci Rep.* (2018) 8:15007. 10.1038/s41598-018-33471-2 30302028 PMC6177478

[49] Zhang M Tang J Li Y Xie Y Shan H Chen M Curcumin attenuates skeletal muscle mitochondrial impairment in COPD rats: Pgc-1alpha/Sirt3 pathway involved. *Chem Biol Interact.* (2017) 277:168–75. 10.1016/j.cbi.2017.09.018 28951138

[50] Buondonno I Sassi F Carignano G Dutto F Ferreri C Pili F From mitochondria to healthy aging: the role of branched-chain amino acids treatment: mater a randomized study. *Clin Nutr.* (2020) 39:2080–91. 10.1016/j.clnu.2019.10.013 31672329

[51] Moro T Brightwell C Phalen D McKenna C Lane S Porter C Low skeletal muscle capillarization limits muscle adaptation to resistance exercise training in older adults. *Exp Gerontol.* (2019) 127:110723. 10.1016/j.exger.2019.110723 31518665 PMC6904952

[52] Biglari S Afousi A Mafi F Shabkhiz F. High-intensity interval training-induced hypertrophy in gastrocnemius muscle via improved IGF-I/Akt/FoxO and myostatin/Smad signaling pathways in rats. *Physiol Int.* (2020) 107:220–30. 10.1556/2060.2020.00020 32644938

[53] Schiaffino S Dyar K Ciciliot S Blaauw B Sandri M. Mechanisms regulating skeletal muscle growth and atrophy. *FEBS J.* (2013) 280:4294–314. 10.1111/febs.12253 23517348

[54] Lautherbach N Goncalves D Silveira W Paula-Gomes S Valentim R Zanon N Urocortin 2 promotes hypertrophy and enhances skeletal muscle function through camp and insulin/Igf-1 signaling pathways. *Mol Metab.* (2022) 60:101492. 10.1016/j.molmet.2022.101492 35390501 PMC9035725

[55] Zhang H Chi M Chen L Sun X Wan L Yang Q Linalool prevents cisplatin induced muscle atrophy by regulating Igf-1/Akt/FoxO pathway. *Front Pharmacol.* (2020) 11:598166. 10.3389/fphar.2020.598166 33390985 PMC7774296

[56] van den Hoek A Zondag G Verschuren L de Ruiter C Attema J de Wit E A novel nutritional supplement prevents muscle loss and accelerates muscle mass recovery in caloric-restricted mice. *Metabolism.* (2019) 97:57–67. 10.1016/j.metabol.2019.05.012 31153978

[57] Meyer A Zoll J Charles A Charloux A de Blay F Diemunsch P Skeletal muscle mitochondrial dysfunction during chronic obstructive pulmonary disease: central actor and therapeutic target. *Exp Physiol.* (2013) 98:1063–78. 10.1113/expphysiol.2012.069468 23377494

[58] Liu W Su W Yang X Bai J Zhong X He Z. [Cigarette smoke extract induces senescence of murine skeletal muscle cells by oxidative stress-induced down-regulation of histone deacetylase 2]. *Chin J Cell Mol Immunol.* (2015) 31:630–3. 25940290

[59] Barreiro E Peinado V Galdiz J Ferrer E Marin-Corral J Sánchez F Cigarette smoke-induced oxidative stress: a role in chronic obstructive pulmonary disease skeletal muscle dysfunction. *Am J Respir Crit Care Med.* (2010) 182:477–88. 10.1164/rccm.200908-1220OC 20413628

[60] Decker S Matias A Bannon S Madden J Alexandrou-Majaj N Layec G. Effects of cigarette smoke on in situ mitochondrial substrate oxidation of slow- and fast-twitch skeletal muscles. *Life Sci.* (2023) 315:121376. 10.1016/j.lfs.2023.121376 36646379

[61] Robison P Sussan T Chen H Biswal S Schneider M Hernandez-Ochoa E. Impaired calcium signaling in muscle fibers from intercostal and foot skeletal muscle in a cigarette smoke-induced mouse model of COPD. *Muscle Nerve.* (2017) 56:282–91. 10.1002/mus.25466 27862020 PMC5426995

[62] Conlon T John-Schuster G Heide D Pfister D Lehmann M Hu Y Inhibition of LTβR signalling activates WNT-induced regeneration in lung. *Nature.* (2020) 588:151–6. 10.1038/s41586-020-2882-8 33149305 PMC7718297

[63] Rom O Kaisari S Aizenbud D Reznick A. Involvement of E3 ubiquitin ligases in cigarette smoke associated muscle catabolism. *Free Radic Biol Med.* (2014) 75:S5. 10.1016/j.freeradbiomed.2014.10.835 26461398

[64] Rom O Kaisari S Aizenbud D Reznick A. Essential amino acid leucine and proteasome inhibitor Mg132 attenuate cigarette smoke induced catabolism in C2 myotubes. *Adv Exp Med Biol.* (2013) 788:25–33. 10.1007/978-94-007-6627-3_4 23835954

[65] Rom O Kaisari S Aizenbud D Reznick A. Cigarette smoke and muscle catabolism in C2 myotubes. *Mech Ageing Dev.* (2013) 134:24–34. 10.1016/j.mad.2012.11.004 23262287

[66] Zhang L Li D Chang C Sun Y. Myostatin/Hif2alpha-mediated ferroptosis is involved in skeletal muscle dysfunction in chronic obstructive pulmonary disease. *Int J Chronic Obstr Pulm Dis.* (2022) 17:2383–99. 10.2147/COPD.S377226 36185172 PMC9519128

[67] Caron M Morissette M Theriault M Nikota J Stampfli M Debigare R. Alterations in skeletal muscle cell homeostasis in a mouse model of cigarette smoke exposure. *PLoS One.* (2013) 8:e66433. 10.1371/journal.pone.0066433 23799102 PMC3682961

[68] Ajime T Serre J Wust R Messa G Poffe C Swaminathan A Two weeks of smoking cessation reverse cigarette smoke-induced skeletal muscle atrophy and mitochondrial dysfunction in mice. *Nicotine Tob Res.* (2021) 23:143–51. 10.1093/ntr/ntaa016 31965191

[69] Kaphalia L Calhoun W. Alcoholic lung injury: metabolic, biochemical and immunological aspects. *Toxicol Lett.* (2013) 222:171–9. 10.1016/j.toxlet.2013.07.016 23892124 PMC3806189

[70] Caceres-Ayala C Pautassi R Acuña M Cerpa W Rebolledo D. The functional and molecular effects of problematic alcohol consumption on skeletal muscle: a focus on athletic performance. *Am J Drug Alcohol Abuse.* (2022) 48:133–47. 10.1080/00952990.2022.2041025 35389308

[71] Moser S Brown A Clark B Arnold W Baumann C. Neuromuscular mechanisms of weakness in a mouse model of chronic alcoholic myopathy. *Alcohol Clin Exp Res.* (2022) 46:1636–47. 10.1111/acer.14907 35869821 PMC9804636

[72] Shenkman B Belova S Zinovyeva O Samkhaeva N Mirzoev T Vilchinskaya N Effect of chronic alcohol abuse on anabolic and catabolic signaling pathways in human skeletal muscle. *Alcohol Clin Exp Res.* (2018) 42:41–52. 10.1111/acer.13531 29044624

[73] Steiner J Lang C. Dysregulation of skeletal muscle protein metabolism by alcohol. *Am J Physiol Endocrinol Metab.* (2015) 308:E699–712. 10.1152/ajpendo.00006.2015 25759394 PMC4420901

[74] Reed C Buhr T Tystahl A Bauer E Clark P Valentine R. The effects of voluntary binge-patterned ethanol ingestion and daily wheel running on signaling of muscle protein synthesis and degradation in female mice. *Alcohol.* (2022) 104:45–52. 10.1016/j.alcohol.2022.06.004 35926812

[75] Otis J Guidot D. Procysteine increases alcohol-depleted glutathione stores in rat plantaris following a period of abstinence. *Alcohol Alcohol.* (2010) 45:495–500. 10.1093/alcalc/agq066 20935073 PMC2981520

[76] Kaluza J Harris H Linden A Wolk A. Alcohol consumption and risk of chronic obstructive pulmonary disease: a prospective cohort study of men. *Am J Epidemiol.* (2019) 188:907–16. 10.1093/aje/kwz020 30877760

[77] Cáceres-Ayala C Mira R Acuña M Brandan E Cerpa W Rebolledo D. Episodic binge-like ethanol reduces skeletal muscle strength associated with atrophy, fibrosis, and inflammation in young rats. *Int J Mol Sci.* (2023) 24:1655. 10.3390/ijms24021655 36675170 PMC9861047

[78] Ma K Huang F Qiao R Miao L. Pathogenesis of sarcopenia in chronic obstructive pulmonary disease. *Front Physiol.* (2022) 13:850964. 10.3389/fphys.2022.850964 35928562 PMC9343800

[79] Zampogna E Paneroni M Belli S Aliani M Gandolfo A Visca D Pulmonary rehabilitation in patients recovering from COVID-19. *Respiration.* (2021) 100:416–22. 10.1159/000514387 33784696 PMC8089404

[80] Rudrappa S Wilkinson D Greenhaff P Smith K Idris I Atherton P. Human skeletal muscle disuse atrophy: effects on muscle protein synthesis, breakdown, and insulin resistance-a qualitative review. *Front Physiol.* (2016) 7:361. 10.3389/fphys.2016.00361 27610086 PMC4997013

[81] Kneppers A Langen R Gosker H Verdijk L Cebron Lipovec N Leermakers P Increased myogenic and protein turnover signaling in skeletal muscle of chronic obstructive pulmonary disease patients with sarcopenia. *J Am Med Dir Assoc.* (2017) 18:637.e1–11. 10.1016/j.jamda.2017.04.016 28578881

[82] Nunes E Stokes T McKendry J Currier B Phillips S. Disuse-induced skeletal muscle atrophy in disease and nondisease states in humans: mechanisms, prevention, and recovery strategies. *Am J Physiol Cell Physiol.* (2022) 322:C1068–84. 10.1152/ajpcell.00425.2021 35476500

[83] de Boer M Selby A Atherton P Smith K Seynnes O Maganaris C The temporal responses of protein synthesis, gene expression and cell signalling in human quadriceps muscle and patellar tendon to disuse. *J Physiol.* (2007) 585:241–51. 10.1113/jphysiol.2007.142828 17901116 PMC2375459

[84] Kilroe S Fulford J Holwerda A Jackman S Lee B Gijsen A Short-term muscle disuse induces a rapid and sustained decline in daily myofibrillar protein synthesis rates. *Am J Physiol Endocrinol Metab.* (2020) 318:E117–30. 10.1152/ajpendo.00360.2019 31743039

[85] Couillard A Koechlin C Cristol J Varray A Prefaut C. Evidence of local exercise-induced systemic oxidative stress in chronic obstructive pulmonary disease patients. *Eur Respir J.* (2002) 20:1123–9. 10.1183/09031936.02.00014302 12449164

[86] Covey M Collins E Reynertson S Dilling D. Resistance training as a preconditioning strategy for enhancing aerobic exercise training outcomes in COPD. *Respir Med.* (2014) 108:1141–52. 10.1016/j.rmed.2014.06.001 24958605 PMC4130772

[87] Phu S Boersma D Duque G. Exercise and sarcopenia. *J Clin Densitom.* (2015) 18:488–92. 10.1016/j.jocd.2015.04.011 26071171

[88] Shi H Scheffler J Pleitner J Zeng C Park S Hannon K Modulation of skeletal muscle fiber type by mitogen-activated protein kinase signaling. *FASEB J.* (2008) 22:2990–3000. 10.1096/fj.07-097600 18417546

[89] Kuro-o M Matsumura Y Aizawa H Kawaguchi H Suga T Utsugi T Mutation of the mouse klotho gene leads to a syndrome resembling ageing. *Nature.* (1997) 390:45–51. 10.1038/36285 9363890

[90] Suga T Kurabayashi M Sando Y Ohyama Y Maeno T Maeno Y Disruption of the klotho gene causes pulmonary emphysema in mice. Defect in maintenance of pulmonary integrity during postnatal life. *Am J Respir Cell Mol Biol.* (2000) 22:26–33. 10.1165/ajrcmb.22.1.3554 10615062

[91] Phelps M Pettan-Brewer C Ladiges W Yablonka-Reuveni Z. Decline in muscle strength and running endurance in klotho deficient C57bl/6 Mice. *Biogerontology.* (2013) 14:729–39. 10.1007/s10522-013-9447-2 24030242 PMC3851892

[92] Benoit B Meugnier E Castelli M Chanon S Vieille-Marchiset A Durand C Fibroblast growth factor 19 regulates skeletal muscle mass and ameliorates muscle wasting in mice. *Nat Med.* (2017) 23:990–6. 10.1038/nm.4363 28650457

[93] Oost L Kustermann M Armani A Blaauw B Romanello V. Fibroblast growth factor 21 controls mitophagy and muscle mass. *J Cachexia Sarcopenia Muscle.* (2019) 10:630–42. 10.1002/jcsm.12409 30895728 PMC6596457

[94] Bres E Bouvier J Courtay A Delaire L Humblot J Cuerq C Fgf19 and muscle architecture in older patients. *Exp Gerontol.* (2023) 174:112120. 10.1016/j.exger.2023.112120 36764368

[95] Bostrom P Wu J Jedrychowski M Korde A Ye L Lo J A Pgc1-Alpha-dependent myokine that drives brown-fat-like development of white fat and thermogenesis. *Nature.* (2012) 481:463–8. 10.1038/nature10777 22237023 PMC3522098

[96] Zhang L Sun Y. Muscle-bone crosstalk in chronic obstructive pulmonary disease. *Front Endocrinol.* (2021) 12:724911. 10.3389/fendo.2021.724911 34650518 PMC8505811

[97] Vaughan R Gannon N Mermier C Conn C. Irisin, a unique non-inflammatory myokine in stimulating skeletal muscle metabolism. *J Physiol Biochem.* (2015) 71:679–89. 10.1007/s13105-015-0433-9 26399516

[98] Sundarrajan L Rajeswari J Weber L Unniappan S. The sympathetic/beta-adrenergic pathway mediates irisin regulation of cardiac functions in zebrafish. *Comp Biochem Physiol Part A Mol Integr Physiol.* (2021) 259:111016. 10.1016/j.cbpa.2021.111016 34126232

[99] Schols A Soeters P Mostert R Pluymers R Wouters E. Physiologic effects of nutritional support and anabolic steroids in patients with chronic obstructive pulmonary disease. A placebo-controlled randomized trial. *Am J Respir Crit Care Med.* (1995) 152:1268–74. 10.1164/ajrccm.152.4.7551381 7551381

[100] Pison C Cano N Cherion C Caron F Court-Fortune I Antonini M Multimodal nutritional rehabilitation improves clinical outcomes of malnourished patients with chronic respiratory failure: a randomised controlled trial. *Thorax.* (2011) 66:953–60. 10.1136/thx.2010.154922 21700760

[101] Jagoe R Engelen M. Muscle wasting and changes in muscle protein metabolism in chronic obstructive pulmonary disease. *Eur Respir J Suppl.* (2003) 46:52s–63s. 10.1183/09031936.03.00004608 14621107

[102] Schols A Soeters P Mostert R Saris W Wouters E. Energy balance in chronic obstructive pulmonary disease. *Am Rev Respir Dis.* (1991) 143:1248–52. 10.1164/ajrccm/143.6.1248 2048807

[103] Schols A Ferreira I Franssen F Gosker H Janssens W Muscaritoli M Nutritional assessment and therapy in COPD: a European respiratory society statement. *Eur Respir J.* (2014) 44:1504–20. 10.1183/09031936.00070914 25234804

[104] LandiFSieberCFieldingRRollandYGuralnikJ. Nutritional intervention in sarcopenia: report from the international conference on frailty and sarcopenia research task force. *J Frailty Aging.* (2018) 7:247–52. 10.14283/jfa.2017.2630298173

[105] Houston D Nicklas B Ding J Harris T Tylavsky F Newman A Dietary protein intake is associated with lean mass change in older, community-dwelling adults: the health, aging, and body composition (health ABC) study. *Am J Clin Nutr.* (2008) 87:150–5. 10.1093/ajcn/87.1.150 18175749

[106] Baum J Kim I Wolfe R. Protein consumption and the elderly: what is the optimal level of intake? *Nutrients.* (2016) 8:359. 10.3390/nu8060359 27338461 PMC4924200

[107] Cruz-Jentoft A. Beta-hydroxy-beta-methyl butyrate (HMB): from experimental data to clinical evidence in sarcopenia. *Curr Protein Peptide Sci.* (2018) 19:668–72. 10.2174/1389203718666170529105026 28554316

[108] Engelen M Wouters E Deutz N Menheere P Schols A. Factors contributing to alterations in skeletal muscle and plasma amino acid profiles in patients with chronic obstructive pulmonary disease. *Am J Clin Nutr.* (2000) 72:1480–7. 10.1093/ajcn/72.6.1480 11101475

[109] Yoneda T Yoshikawa M Fu A Tsukaguchi K Okamoto Y Takenaka H. Plasma levels of amino acids and hypermetabolism in patients with chronic obstructive pulmonary disease. *Nutrition.* (2001) 17:95–9. 10.1016/s0899-9007(00)00509-8 11240335

[110] Atherton P Smith K Etheridge T Rankin D Rennie M. Distinct anabolic signalling responses to amino acids in C2C12 skeletal muscle cells. *Amino Acids.* (2010) 38:1533–9. 10.1007/s00726-009-0377-x 19882215

[111] Bauer J Verlaan S Bautmans I Brandt K Donini L Maggio M Effects of a vitamin D and leucine-enriched whey protein nutritional supplement on measures of sarcopenia in older adults, the provide study: a randomized, double-blind, placebo-controlled trial. *J Am Med Direct Assoc.* (2015) 16:740–7. 10.1016/j.jamda.2015.05.021 26170041

[112] Deutz N Pereira S Hays N Oliver J Edens N Evans C Effect of beta-hydroxy-beta-methylbutyrate (HMB) on lean body mass during 10 days of bed rest in older adults. *Clin Nutr.* (2013) 32:704–12. 10.1016/j.clnu.2013.02.011 23514626

[113] Deutz N Matheson E Matarese L Luo M Baggs G Nelson J Readmission and mortality in malnourished, older, hospitalized adults treated with a specialized oral nutritional supplement: a randomized clinical trial. *Clin Nutr.* (2016) 35:18–26. 10.1016/j.clnu.2015.12.010 26797412

[114] van de Bool C Rutten E van Helvoort A Franssen F Wouters E Schols AA. Randomized clinical trial investigating the efficacy of targeted nutrition as adjunct to exercise training in COPD. *J Cachexia Sarcopenia Muscle.* (2017) 8:748–58. 10.1002/jcsm.12219 28608438 PMC5659064

[115] Marco E Sánchez-Rodríguez D Dávalos-Yerovi V Duran X Pascual E Muniesa J Malnutrition according to ESPEN consensus predicts hospitalizations and long-term mortality in rehabilitation patients with stable chronic obstructive pulmonary disease. *Clin Nutr.* (2019) 38:2180–6. 10.1016/j.clnu.2018.09.014 30342931

[116] Sergi G Coin A Marin S Vianello A Manzan A Peruzza S Body composition and resting energy expenditure in elderly male patients with chronic obstructive pulmonary disease. *Respir Med.* (2006) 100:1918–24. 10.1016/j.rmed.2006.03.008 16635565

[117] Cai B Zhu Y Ma Y Xu Z Zao Y Wang J Effect of supplementing a high-fat, low-carbohydrate enteral formula in COPD patients. *Nutrition.* (2003) 19:229–32. 10.1016/s0899-9007(02)01064-x 12620524

[118] Frankfort J Fischer C Stansbury D McArthur D Brown S Light R. Effects of high- and low-carbohydrate meals on maximum exercise performance in chronic airflow obstruction. *Chest.* (1991) 100:792–5. 10.1378/chest.100.3.792 1889274

[119] Kuo C Shiao G Lee J. The effects of high-fat and high-carbohydrate diet loads on gas exchange and ventilation in COPD patients and normal subjects. *Chest.* (1993) 104:189–96. 10.1378/chest.104.1.189 8325067

[120] Calder P. n-3 PUFA and inflammation: from membrane to nucleus and from bench to bedside. *Proc Nutr Soc.* (2020) 10.1017/s0029665120007077 [Epub ahead of print]. 32624016

[121] Marion-Letellier R Savoye G Ghosh S. Polyunsaturated fatty acids and inflammation. *IUBMB Life.* (2015) 67:659–67. 10.1002/iub.1428 26397837

[122] Cotogni P Muzio G Trombetta A Ranieri V Canuto R. Impact of the omega-3 to omega-6 polyunsaturated fatty acid ratio on cytokine release in human alveolar cells. *JPEN J Parenter Enteral Nutr.* (2011) 35:114–21. 10.1177/0148607110372392 21224438

[123] Broekhuizen R Wouters E Creutzberg E Weling-Scheepers C Schols A. Polyunsaturated fatty acids improve exercise capacity in chronic obstructive pulmonary disease. *Thorax.* (2005) 60:376–82. 10.1136/thx.2004.030858 15860712 PMC1758900

[124] Calder P Laviano A Lonnqvist F Muscaritoli M Öhlander M Schols A. Targeted medical nutrition for cachexia in chronic obstructive pulmonary disease: a randomized, controlled trial. *J Cachexia Sarcopenia Muscle.* (2018) 9:28–40. 10.1002/jcsm.12228 28891198 PMC5803606

[125] Sugawara K Takahashi H Kasai C Kiyokawa N Watanabe T Fujii S Effects of nutritional supplementation combined with low-intensity exercise in malnourished patients with COPD. *Respir Med.* (2010) 104:1883–9. 10.1016/j.rmed.2010.05.008 20627502

[126] Scoditti E Massaro M Garbarino S Toraldo D. Role of diet in chronic obstructive pulmonary disease prevention and treatment. *Nutrients.* (2019) 11:1357. 10.3390/nu11061357 31208151 PMC6627281

[127] de Batlle J Sauleda J Balcells E Gomez F Mendez M Rodriguez E Association between omega3 and omega6 fatty acid intakes and serum inflammatory markers in COPD. *J Nutr Biochem.* (2012) 23:817–21. 10.1016/j.jnutbio.2011.04.005 21889886

[128] Herbst E Paglialunga S Gerling C Whitfield J Mukai K Chabowski A Omega-3 supplementation alters mitochondrial membrane composition and respiration kinetics in human skeletal muscle. *J Physiol.* (2014) 592:1341–52. 10.1113/jphysiol.2013.267336 24396061 PMC3961091

[129] de van der Schueren M Laviano A Blanchard H Jourdan M Arends J Baracos V. Systematic review and meta-analysis of the evidence for oral nutritional intervention on nutritional and clinical outcomes during chemo(radio)therapy: current evidence and guidance for design of future trials. *Ann Oncol.* (2018) 29:1141–53. 10.1093/annonc/mdy114 29788170 PMC5961292

[130] Zhu M Wang T Wang C Ji Y. The association between vitamin D and COPD risk, severity, and exacerbation: an updated systematic review and meta-analysis. *Int J Chronic Obstr Pulm Dis.* (2016) 11:2597–607. 10.2147/COPD.S101382 27799758 PMC5079694

[131] Christakos S Dhawan P Verstuyf A Verlinden L Carmeliet G. Vitamin D: metabolism, molecular mechanism of action, and pleiotropic effects. *Physiol Rev.* (2016) 96:365–408. 10.1152/physrev.00014.2015 26681795 PMC4839493

[132] Valle M Russo C Casabona A Crimi N Crimi C Colaianni V Anti-inflammatory role of vitamin d in muscle dysfunctions of patients with chronic obstructive pulmonary disease: a comprehensive review. *Minerva Med.* (2022) 114:357–71. 10.23736/s0026-4806.22.07879-x 35332756

[133] Carson E Pourshahidi L Madigan S Baldrick F Kelly M Laird E Vitamin D status is associated with muscle strength and quality of life in patients with COPD: a seasonal prospective observation study. *Int J Chronic Obstr Pulm Dis.* (2018) 13:2613–22. 10.2147/copd.S166919 30214179 PMC6118240

[134] Jolliffe D Greenberg L Hooper R Mathyssen C Rafiq R de Jongh R Vitamin D to prevent exacerbations of COPD: systematic review and meta-analysis of individual participant data from randomised controlled trials. *Thorax.* (2019) 74:337–45. 10.1136/thoraxjnl-2018-212092 30630893

[135] Girgis C. Vitamin D and skeletal muscle: emerging roles in development, anabolism and repair. *Calcif Tissue Int.* (2020) 106:47–57. 10.1007/s00223-019-00583-4 31312865

[136] Rafiq R Aleva F Schrumpf J Daniels J Bet P Boersma W Vitamin D supplementation in chronic obstructive pulmonary disease patients with low serum vitamin D: a randomized controlled trial. *Am J Clin Nutr.* (2022) 116:491–9. 10.1093/ajcn/nqac083 35383823 PMC9348978

[137] Mølmen K Hammarström D Pedersen K Lian Lie A Steile R Nygaard H Vitamin D(3) supplementation does not enhance the effects of resistance training in older adults. *J Cachexia Sarcopenia Muscle.* (2021) 12:599–628. 10.1002/jcsm.12688 33788419 PMC8200443

[138] Beaudart C Locquet M Touvier M Reginster J Bruyere O. Association between dietary nutrient intake and sarcopenia in the sarcophage study. *Aging Clin Exp Res.* (2019) 31:815–24. 10.1007/s40520-019-01186-7 30955158

[139] Piscaer I van den Ouweland J Vermeersch K Reynaert N Franssen F Keene S Low vitamin K status is associated with increased elastin degradation in chronic obstructive pulmonary disease. *J Clin Med.* (2019) 8:1116. 10.3390/jcm8081116 31357639 PMC6724066

[140] Shen T Bimali M Faramawi M Orloff M. Consumption of vitamin K and vitamin a are associated with reduced risk of developing emphysema: NHANES 2007-2016. *Front Nutr.* (2020) 7:47. 10.3389/fnut.2020.00047 32391372 PMC7192023

[141] Sha T Li W He H Wu J Wang Y Li H. Causal relationship of genetically predicted serum micronutrients levels with sarcopenia: a Mendelian randomization study. *Front Nutr.* (2022) 9:913155. 10.3389/fnut.2022.913155 35811987 PMC9257254

[142] Altun M Edstrom E Spooner E Flores-Moralez A Bergman E Tollet-Egnell P Iron load and redox stress in skeletal muscle of aged rats. *Muscle Nerve.* (2007) 36:223–33. 10.1002/mus.20808 17503500

[143] Arruda L Arruda S Campos N de Valencia F Siqueira E. Dietary iron concentration may influence aging process by altering oxidative stress in tissues of adult rats. *PLoS One.* (2013) 8:e61058. 10.1371/journal.pone.0061058 23593390 PMC3625229

[144] Hofer T Marzetti E Xu J Seo A Gulec S Knutson M Increased iron content and RNA oxidative damage in skeletal muscle with aging and disuse atrophy. *Exp Gerontol.* (2008) 43:563–70. 10.1016/j.exger.2008.02.007 18395385 PMC2601529

[145] Xu J Knutson M Carter C Leeuwenburgh C. Iron accumulation with age, oxidative stress and functional decline. *PLoS One.* (2008) 3:e2865. 10.1371/journal.pone.0002865 18682742 PMC2481398

[146] Perez-Peiro M Alvarado M Martin-Ontiyuelo C Duran X Rodriguez-Chiaradia D Barreiro E. Iron depletion in systemic and muscle compartments defines a specific phenotype of severe COPD in female and male patients: implications in exercise tolerance. *Nutrients.* (2022) 14:3929. 10.3390/nu14193929 36235581 PMC9571884

[147] Seo M Kim M Park S Rhee E Park C Lee W The association between daily calcium intake and sarcopenia in older, non-obese Korean Adults: the fourth Korea National Health and Nutrition Examination Survey (KNHANES IV) 2009. *Endocr J.* (2013) 60:679–86. 10.1507/endocrj.ej12-0395 23357977

[148] Chen Y Yang K Chang H Lee L Lu C Huang K. Low serum selenium level is associated with low muscle mass in the community-dwelling elderly. *J Am Med Direct Assoc.* (2014) 15:807–11. 10.1016/j.jamda.2014.06.014 25112230

[149] Doorduin J Sinderby C Beck J Stegeman D van Hees H van der Hoeven J The calcium sensitizer levosimendan improves human diaphragm function. *Am J Respir Crit Care Med.* (2012) 185:90–5. 10.1164/rccm.201107-1268OC 21960535

[150] van Hees H Dekhuijzen P Heunks L. Levosimendan enhances force generation of diaphragm muscle from patients with chronic obstructive pulmonary disease. *Am J Respir Crit Care Med.* (2009) 179:41–7. 10.1164/rccm.200805-732OC 18990676

[151] Fodor J Al-Gaadi D Czirjak T Olah T Dienes B Csernoch L Improved calcium homeostasis and force by selenium treatment and training in aged mouse skeletal muscle. *Sci Rep.* (2020) 10:1707. 10.1038/s41598-020-58500-x 32015413 PMC6997352

[152] van Dijk M Dijk F Hartog A van Norren K Verlaan S van Helvoort A Reduced dietary intake of micronutrients with antioxidant properties negatively impacts muscle health in aged mice. *J Cachexia Sarcopenia Muscle.* (2018) 9:146–59. 10.1002/jcsm.12237 29045021 PMC5803605

[153] Vidlar A Vostalova J Ulrichova J Student V Krajicek M Vrbkova J The safety and efficacy of a silymarin and selenium combination in men after radical prostatectomy - a six month placebo-controlled double-blind clinical trial. *Biomed Pap Med Fac Univ Palacky Olomouc Czech Repub.* (2010) 154:239–44. 10.5507/bp.2010.036 21048810

[154] Vostalova J Vidlar A Ulrichova J Vrbkova J Simanek V Student V. Use of selenium-silymarin mix reduces lower urinary tract symptoms and prostate specific antigen in men. *Phytomedicine.* (2013) 21:75–81. 10.1016/j.phymed.2013.07.018 24012146

[155] Darago A Klimczak M Stragierowicz J Stasikowska-Kanicka O Kilanowicz A. The effect of zinc, selenium, and their combined supplementation on androgen receptor protein expression in the prostate lobes and serum steroid hormone concentrations of Wistar rats. *Nutrients.* (2020) 12:153. 10.3390/nu12010153 31935838 PMC7019230

[156] Gouzi F Maury J Héraud N Molinari N Bertet H Ayoub B Additional effects of nutritional antioxidant supplementation on peripheral muscle during pulmonary rehabilitation in COPD patients: a randomized controlled trial. *Oxid Med Cell Longev.* (2019) 2019:5496346. 10.1155/2019/5496346 31178967 PMC6501222

[157] Welch A. Nutritional influences on age-related skeletal muscle loss. *Proc Nutr Soc.* (2014) 73:16–33. 10.1017/S0029665113003698 24229650

[158] Ruljancic N Popovic-Grle S Rumenjak V Sokolic B Malic A Mihanovic M COPD: magnesium in the plasma and polymorphonuclear cells of patients during a stable phase. *COPD.* (2007) 4:41–7. 10.1080/15412550601169513 17364676

[159] Ahmadi A Eftekhari M Mazloom Z Masoompour M Fararooei M Eskandari M Fortified whey beverage for improving muscle mass in chronic obstructive pulmonary disease: a single-blind, randomized clinical trial. *Respir Res.* (2020) 21:216. 10.1186/s12931-020-01466-1 32807165 PMC7430110

[160] Scott D Blizzard L Fell J Giles G Jones G. Associations between dietary nutrient intake and muscle mass and strength in community-dwelling older adults: the Tasmanian older adult cohort study. *J Am Geriatr Soc.* (2010) 58:2129–34. 10.1111/j.1532-5415.2010.03147.x 21054294

[161] Dominguez L Barbagallo M Lauretani F Bandinelli S Bos A Corsi A Magnesium and muscle performance in older persons: the Inchianti study. *Am J Clin Nutr.* (2006) 84:419–26. 10.1093/ajcn/84.1.419 16895893 PMC2669297

[162] Fiaccadori E Coffrini E Ronda N Vezzani A Cacciani G Fracchia C Hypophosphatemia in course of chronic obstructive pulmonary disease. Prevalence, mechanisms, and relationships with skeletal muscle phosphorus content. *Chest.* (1990) 97:857–68. 10.1378/chest.97.4.857 2108845

[163] Fiaccadori E Coffrini E Fracchia C Rampulla C Montagna T Borghetti A. Hypophosphatemia and phosphorus depletion in respiratory and peripheral muscles of patients with respiratory failure due to COPD. *Chest.* (1994) 105:1392–8. 10.1378/chest.105.5.1392 8181325

[164] Layec G Hart C Trinity J Kwon O Rossman M Broxterman R Oxygen delivery and the restoration of the muscle energetic balance following exercise: implications for delayed muscle recovery in patients with COPD. *Am J Physiol Endocrinol Metab.* (2017) 313:E94–104. 10.1152/ajpendo.00462.2016 28292763 PMC6109703

[165] Marchesani F Valerio G Dardes N Viglianti B Sanguinetti C. Effect of intravenous fructose 1,6-diphosphate administration in malnourished chronic obstructive pulmonary disease patients with chronic respiratory failure. *Respiration.* (2000) 67:177–82. 10.1159/000029483 10773790

[166] Passey S Hansen M Bozinovski S McDonald C Holland A Vlahos R. Emerging therapies for the treatment of skeletal muscle wasting in chronic obstructive pulmonary disease. *Pharmacol Ther.* (2016) 166:56–70. 10.1016/j.pharmthera.2016.06.013 27373503

[167] Marillier M Bernard A Verges S Neder J. Locomotor muscles in COPD: the rationale for rehabilitative exercise training. *Front Physiol.* (2019) 10:1590. 10.3389/fphys.2019.01590 31992992 PMC6971045

[168] Pan L Wang M Xie X Du C Guo Y. Effects of anabolic steroids on chronic obstructive pulmonary disease: a meta-analysis of randomised controlled trials. *PLoS One.* (2014) 9:e84855. 10.1371/journal.pone.0084855 24427297 PMC3888411

[169] Baillargeon J Urban R Zhang W Zaiden M Javed Z Sheffield-Moore M Testosterone replacement therapy and hospitalization rates in men with COPD. *Chronic Respir Dis.* (2019) 16:1479972318793004. 10.1177/1479972318793004 30205698 PMC6302963

[170] Creutzberg E Wouters E Mostert R Pluymers R Schols A. A role for anabolic steroids in the rehabilitation of patients with COPD? A double-blind, placebo-controlled, randomized trial. *Chest.* (2003) 124:1733–42. 10.1378/chest.124.5.1733 14605042

[171] Kim J Roberts K Dunlap G Perry R Washington T Wolchok J. Nandrolone supplementation does not improve functional recovery in an aged animal model of volumetric muscle loss injury. *J Tissue Eng Regener Med.* (2022) 16:367–79. 10.1002/term.3286 35113494

[172] Maltais F Decramer M Casaburi R Barreiro E Burelle Y Debigare R An official American thoracic society/European respiratory society statement: update on limb muscle dysfunction in chronic obstructive pulmonary disease. *Am J Respir Crit Care Med.* (2014) 189:e15–62. 10.1164/rccm.201402-0373ST 24787074 PMC4098112

[173] Pape G Friedman M Underwood L Clemmons D. The effect of growth hormone on weight gain and pulmonary function in patients with chronic obstructive lung disease. *Chest.* (1991) 99:1495–500. 10.1378/chest.99.6.1495 2036835

[174] Rolland Y Onder G Morley J Gillette-Guyonet S Abellan van Kan G Vellas B. Current and future pharmacologic treatment of sarcopenia. *Clin Geriatr Med.* (2011) 27:423–47. 10.1016/j.cger.2011.03.008 21824556

[175] Collden G Tschop M Muller T. Therapeutic potential of targeting the ghrelin pathway. *Int J Mol Sci.* (2017) 18:798. 10.3390/ijms18040798 28398233 PMC5412382

[176] Rooks D Praestgaard J Hariry S Laurent D Petricoul O Perry R Treatment of sarcopenia with bimagrumab: results from a phase II, randomized, controlled, proof-of-concept study. *J Am Geriatr Soc.* (2017) 65:1988–95. 10.1111/jgs.14927 28653345

[177] Polkey M Praestgaard J Berwick A Franssen F Singh D Steiner M Activin type II receptor blockade for treatment of muscle depletion in chronic obstructive pulmonary disease. A randomized trial. *Am J Respir Crit Care Med.* (2019) 199:313–20. 10.1164/rccm.201802-0286OC 30095981 PMC6363975

[178] van Beers M Rutten-van Mölken M van de Bool C Boland M Kremers S Franssen F Clinical outcome and cost-effectiveness of a 1-year nutritional intervention programme in COPD patients with low muscle mass: the randomized controlled NUTRAIN trial. *Clin Nutr.* (2020) 39:405–13. 10.1016/j.clnu.2019.03.001 30954363

